# Physical Properties and Release Profiles of Chitosan Mixture Films Containing Salicin, Glycerin and Hyaluronic Acid

**DOI:** 10.3390/molecules28237827

**Published:** 2023-11-28

**Authors:** Katarzyna Lewandowska, Alina Sionkowska, Marzanna Kurzawa

**Affiliations:** 1 Department of Biomaterials and Cosmetic Chemistry, Faculty of Chemistry, Nicolaus Copernicus University in Torun, Gagarin 7 Street, 87-100 Torun, Poland; alinas@umk.pl; 2 Department of Analytical Chemistry and Applied Spectroscopy, Faculty of Chemistry, Nicolaus Copernicus University in Torun, Gagarin 7 Street, 87-100 Torun, Poland; jmk@umk.pl

**Keywords:** salicin, chitosan, release process, surface properties, hyaluronic acid

## Abstract

Chitosan (CS) has gained considerable attention due to its distinctive properties and its broad spectrum of potential applications, spanning cosmetics, pharmaceuticals, and biomedical uses. In this study, we characterized thin films comprising chitosan mixtures containing salicin (SAL) and glycerin (GLY), both with and without hyaluronic acid (HA) as active ingredients. Characterization was achieved through release studies of SAL, infrared spectroscopy, microscopy techniques (AFM and SEM), and thermogravimetric analysis (TGA). CS/GLY/SAL and CS/GLY/SAL/HA mixture films were fabricated using the solvent evaporation technique. We probed interactions between the components in the chitosan mixtures via infrared analysis. The concentration of released salicin was monitored at various time intervals in a phosphate buffer (PBS) at pH 5.5 using HPLC. The linear regression analysis for the calibration graph showed a good linear relationship (R^2^ = 0.9996) in the working concentration range of 5–205 mg/dm^3^. Notably, the release of SAL reached its peak after 20 min. Furthermore, the introduction of HA caused changes in the films’ morphology, but their roughness remained largely unchanged. The results obtained were compared, indicating that the release of SAL in the CS mixture films is sufficient for diverse applications, including wound-healing materials and cosmetic beauty masks.

## 1. Introduction

**Figure 1:**
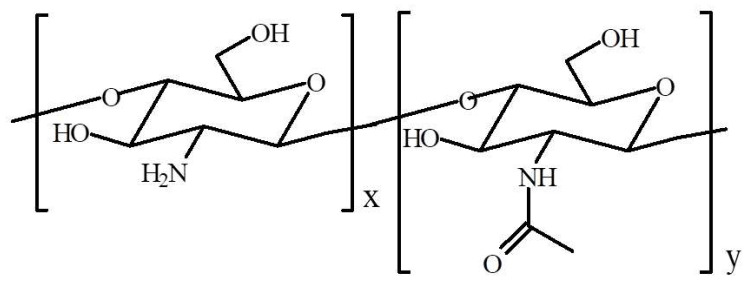
Structure of chitosan.

Natural polymers are extensively used in the cosmetic industry as raw materials and show great potential for biomedical applications due to their film-forming ability, controlled bioactivity, biocompatibility, and natural origin [1,2,3]. In the huge group of biopolymers, the most widely used in cosmetic preparations are polysaccharides and proteins. The above-mentioned macromolecules are not only used because of their possibility of rheology modification but also in several cases as active agents. Chitosan (CS) is a polysaccharide and the main derivative of chitin, commonly found in the exoskeletons of crustaceans and insects. It is a copolymer of glucosamine and N-acetylglucosamine connected by a β (1-4) linkage (Figure 1) [4,5,6].

CS is typically obtained from chitin through a process of partial deacetylation, which is commonly carried out in an alkaline environment [7,8,9]. Chitin, known as the second most abundant polymer in nature after cellulose, has an estimated annual production of approximately 10^10^ to 10^11^ tons [10]. Chitin is commonly found in the exoskeletons of crustaceans and insects, as well as in the cell walls of certain fungi and microorganisms. Thus, chitin holds great importance as a resource due to its abundance and renewability. It has the potential to significantly contribute to the development of sustainable processing methods for bioactive and biodegradable materials. However, despite its abundance, chitin’s inherent insolubility presents challenges for subsequent processing. On the other hand, CS readily dissolves in dilute acidic solutions with a pH below 6.3 using popular organic and inorganic acids, such as formic acid, acetic acid, lactic acid, and hydrochloric acid, which are harmless to humans and the environment. In addition, the kind of acid applied significantly affects the physicochemical properties of CS solutions and other forms, such as films and hydrogels. Furthermore, CS forms water-soluble salts when exposed to aqueous acidic solutions [11,12,13]. CS possesses numerous important features, including film-forming ability, bioactivity, non-toxicity towards humans, biodegradability, and applicability in the fabrication of biomaterials. Therefore, CS finds extensive use in pharmaceutical, biomedical, and cosmetic applications [7,12,14,15,16,17].

**Figure 2:**
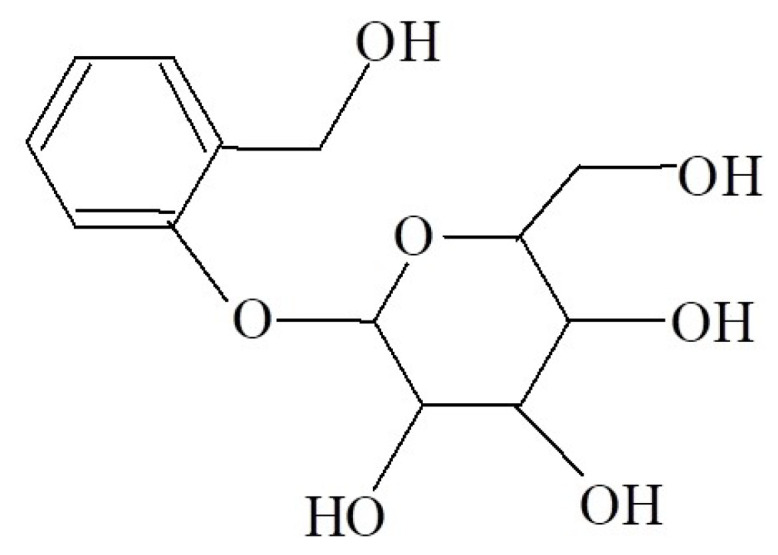
Structure of d-(−)-Salicin.

Polysaccharides have become essential in the development of active substance delivery technology, as they enable the controlled release of active agents in consistent doses over specific timeframes [18,19,20]. CS can serve as a versatile platform for the development of materials in cosmetic and biomedical applications that incorporate various active substances. By blending CS with active agents, such as flavonoids, phenolic acids, vitamins, antioxidants, antibacterial agents, and other biologically active compounds, it becomes feasible to create materials with customized properties. This includes enhancing processability for producing innovative gels, films, emulsions, and materials that find application in wound healing and other biomedical or cosmetics contexts. This approach offers a cost-effective and time-efficient method for generating materials with unique properties. Consequently, the blending of CS and active agents to produce multifunctional, biodegradable blends is an active area of research, with a focus on fabricating a wide range of materials for diverse applications, such as gels, microcapsules, beads, hydrogels, and films. Various reports have studied materials derived from the combination of CS and other active substances [21,22,23]. For example, sensitive hydrogels using CS and hyaluronic acid loaded with the active substance dihydromyricetin have been proposed as potential candidates for guiding skin repair [21]. Liu et al. [22] prepared a series of bilayer films formulated from chitosan, bacterial cellulose, and pullulan as the matrix, with the addition of curcumin at different concentrations as the active compound. Furthermore, Zhu et al. successfully obtained microcapsules based on a chitosan derivative and tree essential oil, demonstrating good thermal stability and antibacterial properties [23]. In this study, we prepared and characterized CS-based films containing salicin and hyaluronic acid.

d-(−)-Salicin (SAL), also known as 2-(hydroxymethyl)phenyl (β-d-glucopyranoside), is an alcoholic beta-glycoside derived from willow bark and leaves (Figure 2) [24,25].

SAL is a white, smooth, water-soluble powder. Willow bark extract is widely recognized for its anti-inflammatory and antiseptic properties [26,27,28]. SAL, the primary component of willow bark extract, undergoes metabolic conversion to salicylic acid within the body and is widely regarded as the key contributor to its anti-inflammatory and analgesic effects [28,29]. Previous studies have provided evidence that when SAL is taken orally, it acts as an effective anti-inflammatory agent. Furthermore, salicin has shown promise in terms of its potential anti-aging effects when applied topically to the skin. Reports suggest that the topical application of salicin can help minimize visible signs of skin aging [29,30,31].

Moreover, various studies described in the literature have shown that, due to its antimicrobial and anti-biofilm properties, salicin can be used in dressing materials as a natural drug in wound-dressing systems with intrinsic antibacterial activity, in the form of hydrogels or films [32,33]. Salicin and its derivatives are excellent cures for skin diseases and infections. Regular external application effectively reduces wrinkles and sagging of the skin and muscles after wounds have been repaired. They also protect the skin from bacterial and fungal infections [34].

Another active substance used in cosmetics and biomedical applications is hyaluronic acid (HA). HA, well known as a natural moisturizing factor, is a natural glycosaminoglycan that participates in cell proliferation, cell migration, and angiogenesis, and it plays an important regulatory role in wound healing [21]. Our previous reports showed that a small addition of HA to CS films improves the performance and physicochemical properties of the prepared films [31,35]. Furthermore, lactic acid is utilized in the formulation of liquid products. Lactic acid (LA) is commonly employed in cosmetic products to regulate the pH of the formulations, and it is a safe substance in cosmetics. Thus, LA is used in pharmaceutical and cosmetic formulations for the fabrication of hygiene and aesthetic products, owing to its moisturizing, antimicrobial, and rejuvenating effects on the skin.

The objective of this study was to develop and evaluate the physical properties and release characteristics of chitosan mixtures formed using a lactic acid solution that incorporates SAL, glycerin, and hyaluronic acid. The utilization of CS films with SAL and HA as active substances presents a novel approach to enhance skin penetration and modify properties, such as texture, hydration, and elasticity. We anticipate that the combination of the aforementioned components will yield a chitosan mixture with physical properties and a salicin release profile that is appealing for both cosmetic and biomedical applications. We will substantiate this claim in the Section 2.

## 2. Results and Discussion

### 2.1. Release Process of Salicin

**Figure 3:**
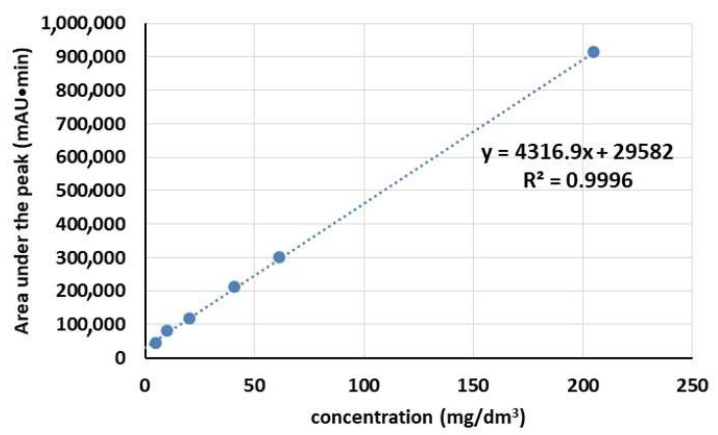
The calibration graph for determination of SAL.

The release process of SAL from the CS materials was investigated in a phosphate buffer (PBS) at pH 5.5, similar to the pH of human skin. Quantitative analysis of SAL in the tested samples was conducted using the standard method, which involves employing a standard curve. Figure 3 presents the calibration graph for determining SAL concentration, which is based on six calibration points. As depicted in Figure 3, a linear calibration curve (R^2^ = 0.9996) was obtained within the concentration range of 5–205 mg/dm^3^.

**Figure 4:**
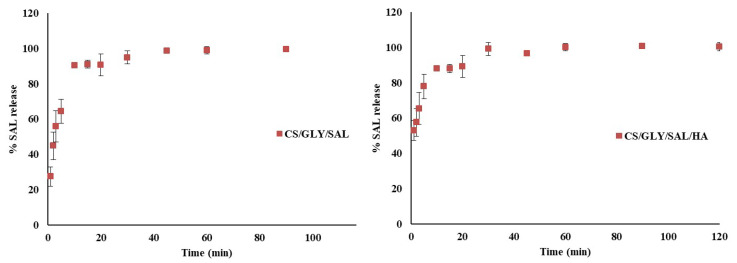
The release profile of SAL from CS mixture films (CS/GLY/SAL and CS/GLY/SAL/HA) into the phosphate buffer (pH 5.5) at 35.5 ± 1 °C.

The release profiles of salicin from two types of CS films (CS/GLY/SAL and CS/GLY/SAL/HA) are depicted in Figure 4.

Within the first 5 min, all the films exhibited an exceptionally rapid release behavior (ranging from 64.40% to 77.78%). This behavior can be attributed to the high solubility of the active substance adhered to the thin film surface in a PBS solution with a pH of 5.5. The maximum release was observed after 20 min. The average percentage of release from the CS/GLY/SAL film was 90.72 ± 6.22%, while for the CS/GLY/HA/SAL films, it amounted to 94.46 ± 7.36%. This behavior can be explained by the formation of a hydrogel, resulting from water absorption and the dissolution of the chitosan matrix (in the form of chitosan lactate) in the PBS solution during the release study of SAL from CS films. Additionally, these observations are in line with our previous research on CS films containing rutin [31]. As outlined in our previous report, the mechanical properties of the CS/GLY/HA films indicate that they are sufficiently elastic to be used as cosmetic beauty masks. The presence of salicin on the film’s surface is advantageous for skin applications in cosmetic masks or wound-healing materials. The active substance (SAL) can be released upon skin application, potentially modifying properties, such as texture, hydration, and elasticity, or promoting a healing effect. As shown in previous reports [32,33,34], regular external application of salicin and its derivatives effectively reduces wrinkles and sagging of the skin and muscles after wounds have been repaired. They also protect the skin from bacterial and fungal infections. In our research, we found that the maximum release of SAL occurred after 20 min in a PBS solution at pH 5.5 for both CS/GLY/SAL and CS/GLY/SAL/HA films. This suggests that beauty masks, if produced using our procedure, should be kept on the skin for at least 20 min. Thus, the characterized CS mixture films with SAL may be promising and attractive materials for cosmetic uses.

### 2.2. Infrared Spectroscopy

**Figure 5:**
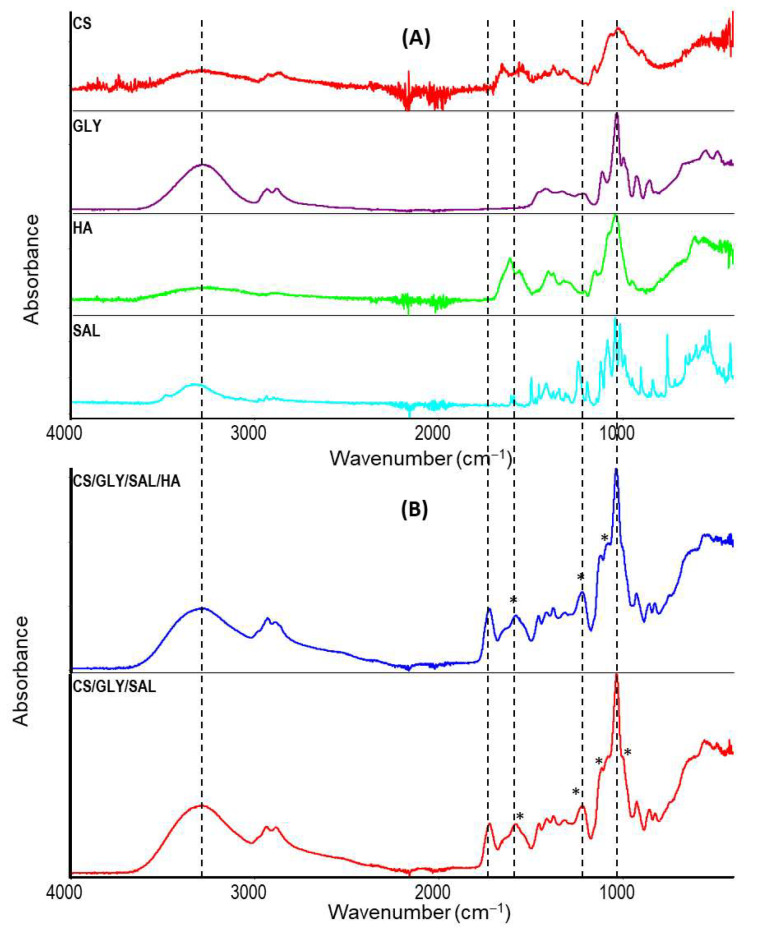
ATR-FTIR spectra of (**A**) pure components and (**B**) CS mixture films: CS/GLY/SAL film (red line), and CS/GLY/SAL/HA (blue line), asterisks (*) indicate changes in the characteristic bands.

The chemical structural changes in the CS films (CS/GLY/SAL and CS/GLY/SAL/HA) were analyzed using infrared spectroscopy in ATR mode. The corresponding infrared spectra of pure components and films are depicted in Figure 5. CS and HA are both hydrophilic polysaccharides, which results in their yielding similar spectra in infrared spectroscopy.

In CS films cast from lactic acid, the following bands were observed: the -OH and -NH stretching vibrations at 3290 cm^−1^, the -CH stretching vibrations at 2930 cm^−1^, the amide I band at 1643 cm^−1^, the amide II band at 1574 cm^−1^, the amide III band at 1457 and 1375 cm^−1^, the β-1,4-glycoside bond at 1118 cm^−1^, and the C-O-C, C-O, C-OH bands at 1082 and 1032 cm^−1^ [35,36]. Additionally, the bands at 1125, 1222, and 1723 cm^−1^ are characteristic of lactic acid, confirming its presence in the CS films [37,38]. Thus, the spectra of CS/GLY/SAL and CS/GLY/SAL/HA films exhibited characteristic bands for their respective components, confirming the formation of the blend films After mixing the components and obtaining the films, the maximum characteristic bands shifted to lower wavenumbers, underwent changes in shape, and became wider (Figure 5B, marked with an asterisk and dotted line). Significant differences in the shape and intensity of bands between 900 and 1700 cm^−1^ were observed compared to pure components, such as CS, SAL, and HA. This observation suggests the potential formation of novel hydrogen bonds between the molecules in the CS formulation. These bonds may form between the amino and hydroxyl groups in CS molecules and the hydroxyl groups of SAL, as well as between the hydroxyl and carboxylic groups of HA and the hydroxyl groups within the CS matrix. The establishment of hydrogen bonds between different molecules may compete with the formation of bonds among molecules of the same type. It is worth noting that the CS films also included GLY are used to produce flexible films suitable for cosmetic purposes and to enhance skin moisturization following topical applications [31,39].

### 2.3. Morphology

**Figure 6:**
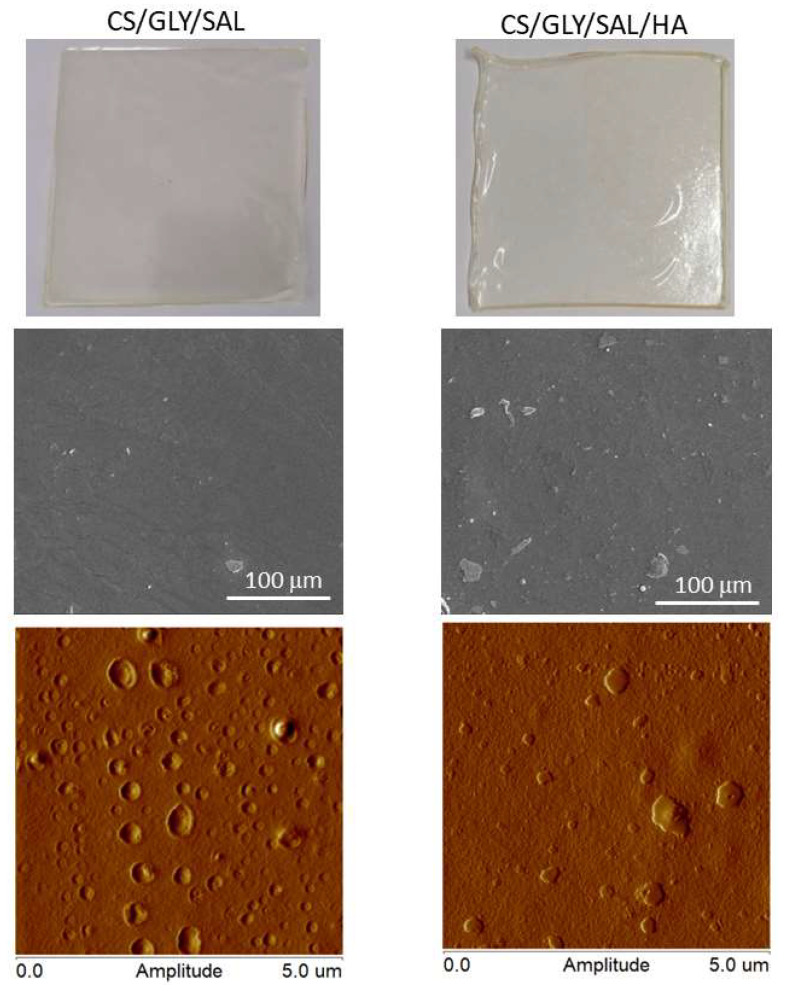
Physical appearances and morphology of film surfaces of various compositions: CS/GLY/SAL (**left panel**) and CS/GLY/SAL/HA (**right panel**).

**Figure 7:**
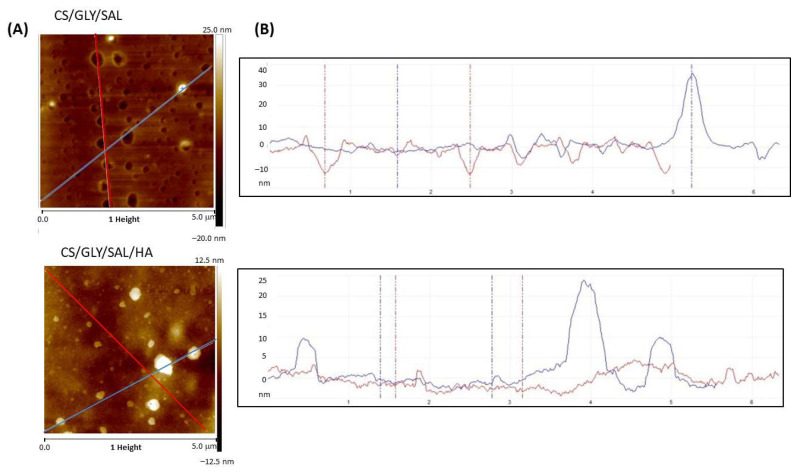
2D **AFM** height images (**A**) and cross-sections along the blue and red lines shown in (**A**) of various compositions (**B**).

The film’s morphology was examined using scanning electron microscopy (SEM) and atomic force microscopy (AFM). The physical characteristics, surface morphology, and topography of the CS films are depicted in Figure 6 and Figure 7. In summary, these films appeared transparent, clear, or pale yellow, and exhibited a high degree of flexibility. As evident from the SEM images, the CS/GLY/SAL film displayed a relatively dense and uniform structure with a flat surface. In contrast, the CS/GLY/SAL/HA film exhibited a rough surface with the presence of a few particles or domains. This phenomenon can be attributed to the drying process, condensation, and repositioning of HA, which resulted in the formation of aggregates or their in situ association with the CS substrates.

It is well known that the AFM method provides images of the surface morphology of the films and measures roughness parameters that help determine the film’s adhesion properties to other substances or tissues. Thus, the AFM images reveal more pronounced differences in the surface topography of the CS films.

In the case of the CS/GLY/SAL film, the film’s image exhibits a pitted surface, with holes evenly distributed across the film surface. On the other hand, the topography of the CS/GLY/SAL/HA film shows a distinct island-like structure. Figure 7B presents a cross-section, illustrating the pits and hills on the film surface. As can be observed, the surface of the CS/GLY/SAL film is dominated by depressions with no prominent hills. In contrast, for the CS/GLY/SAL/HA film, there are only a few hills present on the surface.

The roughness parameter values calculated from AFM images are provided in Table 1. Notably, despite the variations in the morphology of the film surfaces, the resulting R_q_ and R_a_ values are quite similar. Table 1 also shows the thickness of the films, which is slightly greater in the case of the CS/GLY/SAL/HA film. From a cosmetic point of view, the roughness parameters may influence the adhesion of the material to human skin. It appears that both films prepared in this study exhibit similar levels of roughness, falling into the category of non-high-roughness films. Consequently, they may demonstrate comparable adhesion to the skin, a quality crucial for cosmetic applications, particularly when the material is utilized in the form of a mask. In the field of biomedical applications, the surface roughness of samples is also highly significant. However, in this context, much higher surface roughness values are required to correspond to larger surface areas. This characteristic becomes particularly advantageous for the material’s performance in cell culture applications, such as tissue engineering or material constructs [40].

### 2.4. Thermogravimetric Analysis

**Figure 8:**
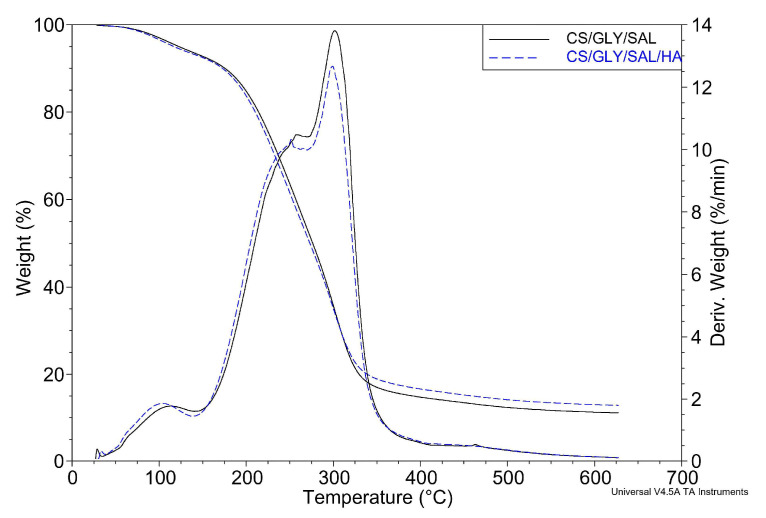
TGA thermograms of CS mixture films: CS/GLY/SAL (solid black line) and CS/GLY/SAL/HA (blue dashed line).

The thermal stability of CS/GLY/SAL and CS/GLY/SAL/HA films can be assessed by examining their mass loss profiles and degradation rates at high temperatures, as reflected in the thermal gravimetry (TG) and differential thermal gravimetry (DTG) curves, as shown in Figure 8. In line with our previous reports [31,41], we can distinguish between two and three mass loss processes for CS and CS/GLY cast from lactic acid, respectively.

For the pure CS films, the initial mass loss of approximately 7% (*w*/*w*) can be attributed to water loss, while the subsequent step, starting at around 200 °C (with T_max_ = 296 °C), corresponds to CS decomposition [41]. In the case of CS/GLY films [31], during the first step (30–130 °C), a weight loss of 6.5% (*w*/*w*) was observed due to the loss of water molecules. Between 140 °C and 270 °C, a weight loss of 45.6% (*w*/*w*) was noted, which can be attributed to the decomposition of GLY and lactic acid. In the third step, the decomposition of CS occurred at 295 °C (with a weight loss of 30% (*w*/*w*)).

In the case of CS films containing SAL (Figure 8), three distinct steps are also observed. Notably, the process of water elimination began at a higher temperature for the CS/GLY/SAL film, with T_max_ values of 110 °C for CS/GLY/SAL and 101 °C for CS/GLY/SAL/HA. The T_max_ values for the second and third steps of the CS films with SAL were comparable, but the peaks for the CS/GLY/SAL film were the highest. For the second step, T_max_ was 256 °C for CS/GLY/SAL and 247 °C for CS/GLY/SAL/HA. This may indicate that the CS/GLY/SAL film exhibits greater thermal stability. These differences may be related to the absence of HA in the CS film, which influences the structural materials, as shown in morphological observations. According to our previous reports [31,35,39], in blends containing CS and HA where they coexist, the properties are strongly influenced by electrostatic interactions that determine the shape of the macromolecules. In aqueous solutions of low ionic strength (when HA is dissolved in water or acetic acid solution), we observed a complete precipitation of the polymeric components from the solution. This is attributed to the strongest electrostatic interactions and/or repulsive forces between the components in the blend. Therefore, in the case of these blends, sodium chloride solutions or HCl aqueous solutions should be used as a solvent when the ionic strength is high enough to prevent the polymer chains from exhibiting polyelectrolyte effects. In this study, we utilized lactic acid, which is stronger than acetic acid, and incorporated a small amount of HA. As a result, precipitation was not observed. However, it is important to note that the addition of HA did have an impact on the morphology and thermal properties of CS films.

## 3. Materials and Methods

### 3.1. Materials

CS powder with a viscosity average molecular weight of 1130 kg/mol and a degree of deacetylation of 75%, lactic acid (LA, 85%, C_3_H_6_0_3_), and d-(−)-Salicin (SAL, ≥99% GC) were purchased from Sigma-Aldrich (St. Louis, MO, USA). Hyaluronic acid (HA, ultra-low molecular weight, a viscosity average molecular weight of 8 kg/mol) was of cosmetic grade, and its structure was confirmed by the infrared spectrum. The viscosity average molecular weight of CS and HA was determined through their intrinsic viscosity using the Mark–Houwink equation (viscometric method) [42]. For CS in 0.1 mol/L acetic acid/0.2 mol/L NaCl, the following constants were observed: K = 1.81 × 10^−3^ mL/g and a = 0.93 at 25 °C [43]. HA in 0.1 mol/L NaCl exhibited the following constant: K = 3.36 × 10^−2^ mL/g and a = 0.79 at 25 °C [44].

All chemicals were applied without further purification.

### 3.2. Preparation of CS Films

CS was dissolved in 0.3 mol/L lactic acid at a concentration of 2% *w*/*v*. The obtained CS solution was blended with other components, including GLY, SAL, and HA, forming two kinds of films: CS/GLY/SAL and CS/GLY/SAL/HA. Table 2 presents the composition of the liquid formulations. GLY liquid was blended with CS solution in a 0.5:1.0 ratio to that of CS powder. SAL powder and HA solution (1% *w*/*v*) were added to the polymer solution at 1% *w*/*w* relative to polymer mass and magnetically stirred (100 rpm) for 24 h at room temperature.

The resulting formulations were poured into square plastic Petri dishes (100 × 100 × 15 mm) and allowed to dry at room temperature under conditions of 40–50% humidity for a period of 72 h. We peeled off the films and applied them for further analysis. Film thickness was determined using a handheld micrometer to the nearest 0.001 mm. Measurements were taken in at least seven random locations of each film, and values were reported as mean ± standard deviation (SD).

### 3.3. SAL Release Behavior

The SAL release characteristics of CS films in phosphate buffer (PBS: NaH_2_PO_4_, Na_2_HPO_4_; pH = 5.5) were investigated. The film samples (~0.23 g) were cut into 4 cm^2^ (length × width) = 2 cm × 2 cm pieces, analytically weighted and then placed in 50 mL of PBS solution and magnetically stirred at 35.5 ± 1 °C. Then, 1.5 mL of the release solution was collected at predetermined intervals (1; 3; 5; 10; 15; 30; 45; 60; 90; and 120 min), and an equal amount of fresh PBS was added as a supplement. The release amount of SAL was determined via high-performance liquid chromatography (UHPLC Shimadzu, Kyoto, Japan) with column 250/4.6 Nucleoshel RP 18.5 μm at a wavelength of 270 nm. The release rate of SAL from films was calculated as follows [23,45]:

(1) $$\%SAL\;release=\frac{M_{t}}{M_{0}}\times 100\%$$

where *M_t_* is the amount of SAL released at different times, and *M_0_* is the amount of SAL in the films.

### 3.4. Infrared Spectroscopy

Infrared spectroscopy was applied to determine the chemical structures and interactions between components in the prepared films. FTIR spectra of films were recorded using a spectrometer (Nicolet iS10, Thermo Fisher Scientific, Waltham, MA, USA) attached with an attenuated total reflectance mode (iDe-Ge-ATR) assembly. The spectral range was 4000–400 cm^−1^. One hundred scans were taken with spectral resolution at 2 cm^−1^. The ATR-FTIR spectra of all samples were analyzed using Omnic 9.3.30 software (Thermo Fisher Scientific, USA).

### 3.5. Morphological Properties

The morphologies of the film surface were investigated using a scanning electron microscope (SEM, Quanta 3D FEG, D9399, FEI, Eindhoven, The Netherland). The films were coated with silver to provide a conductive surface for electron beam interaction.

An atomic force microscope (Nanoscope IIIa Multimode Scanning Probe Microscope, Digital Instruments, Veeco Metrology Group, Santa Barbara, CA, USA) was used to analyze the surface images, operating in tapping mode at room temperature in an air atmosphere. The roughness parameters were determined from AFM images (5 μm × 5 μm) as the root mean square (R_q_) and the arithmetic mean deviation of registered profile (R_a_) using NanoScope Analysis v1.40 software (Bruker, Ettlingen, Germany).

### 3.6. Thermogravimetric Analysis

The thermal stability and degradation of CS films were measured using an SDT 2960 Simultaneous TGA-DTA analyzer from TA Instruments (TA Instruments Manufactures, Eschborn, Germany) over a temperature range from 20 °C to 600 °C at a heating rate of 20 °C/min under a nitrogen atmosphere.

## 4. Conclusions

In this study, we prepared and characterized CS films that incorporated both GLY and SAL, with and without the addition of HA. The inclusion of SAL and HA into the CS matrix resulted in structural modifications within the films, primarily due to the formation of hydrogen bonds. Furthermore, it is worth noting that the CS/GLY/SAL film exhibited greater thermal stability compared to the CS/GLY/SAL/HA film. This may be related to the interactions between CS and HA. Both biopolymers are polyelectrolytes, and their properties, are significantly affected by electrostatic interactions in the solution used for film preparation.

The release kinetics revealed that the maximum release occurred after 20 min in a PBS solution at pH 5.5 for both CS/GLY/SAL and CS/GLY/SAL/HA films. The presence of salicin on the surface of the CS films holds promise for various applications in cosmetics, such as facial masks or wound-healing materials. The observed morphological and physical properties of the CS films containing SAL and HA suggest their potential as a vehicle for delivering SAL as an active substance. Additionally, other components used, such as GLY and lactic acid, are safe and suitable for cosmetic formulations.

## Figures and Tables

**Table 1:** Roughness parameters (R_q_ and R_a_) and thickness (Th) for films with various compositions.

Sample	R_q_ (nm)	R_a_ (nm)	Th (mm)
CS/GLY/SAL	2.84 ± 0.12	1.90 ± 0.10	0.063 ± 0.002
CS/GLY/SAL/HA	2.86 ± 0.30	1.93 ± 0.28	0.070 ± 0.004

**Table 2:** Components of CS-based liquid formulations.

Components	CS/GLY/SAL (% *w*/*w*)	CS/GLY/SAL/HA (% *w*/*w*)
CS	1.95	1.91
GLY	2.19	2.31
SAL	0.02	0.02
HA	0.00	0.02
LA	2.63	2.60
water	to 100	to 100

## Data Availability

All data are shown in the paper.
